# Eclampsia and Neonatal Loss in a Patient With Primary Antiphospholipid Syndrome, Factor V Leiden, and Methylenetetrahydrofolate Reductase (MTHFR) Mutations: A Rare Thrombophilic Triad

**DOI:** 10.7759/cureus.91127

**Published:** 2025-08-27

**Authors:** Sara Hachem, Oussama Fakih, Sally Abi Dargham, Ouidade Aitisha Tabesh, Fouad Fayad

**Affiliations:** 1 Department of Internal Medicine, Lebanese University Faculty of Medicine, Beirut, LBN; 2 Department of Rheumatology, Lebanese Hospital Geitaoui - University Medical Center (UMC), Beirut, LBN; 3 Department of Rheumatology, Lebanese University Faculty of Medicine, Beirut, LBN; 4 Division of Rheumatology, Lebanese American University School of Medicine, Beirut, LBN

**Keywords:** anti-phospholipid antibody syndrome (aps), eclampsia, factor v leiden, mthfr mutation, thrombophilia

## Abstract

Antiphospholipid syndrome (APS) is a systemic autoimmune disorder characterized by an increased risk of thrombosis and pregnancy complications, including placental insufficiency, preeclampsia, and fetal loss. The coexistence of APS with both factor V Leiden and methylenetetrahydrofolate reductase (MTHFR) mutations is exceptionally rare and may further elevate the risk of adverse outcomes. A 23-year-old primigravida developed eclampsia at eight months’ gestation and underwent an emergency cesarean section; unfortunately, her newborn died a few days later. Laboratory evaluation confirmed APS with a positive lupus anticoagulant, anticardiolipin, and anti-β2 glycoprotein I, as well as the presence of factor V Leiden and MTHFR mutations. Hydroxychloroquine, aspirin, and low-molecular-weight heparin therapy were initiated. This case should encourage physicians to broaden their initial screening laboratory tests and consider implementing more comprehensive testing in patients who present with early and severe pre-eclampsia.

## Introduction

Thromboembolic events arise from underlying inherited or acquired conditions. Antiphospholipid syndrome (APS) is an autoimmune disorder that can manifest as arterial and/or venous thrombosis and is a known cause of pregnancy-related morbidity. Antiphospholipid antibodies include lupus anticoagulant, anticardiolipin, and anti-β2 glycoprotein I antibodies [1]. Factor V Leiden mutation is an inherited thrombophilia that leads to resistance to activated protein C, thereby impairing its anticoagulant role and significantly increasing the risk of venous thromboembolism (VTE), particularly in homozygous individuals [2]. Additionally, methylenetetrahydrofolate reductase (MTHFR) deficiency, especially the C677T polymorphism, leads to elevated homocysteine levels by inhibiting its conversion into methionine. Elevated homocysteine causes endothelial dysfunction by damaging blood vessels, thus promoting a prothrombotic state [3].

The coexistence of APS with either factor V Leiden or MTHFR mutations has been previously described. A study by Diz-Kucukkaya et al. reported a prevalence of factor V Leiden in APS cohorts at 10% [4], while another study by Reshetniak et al. found that nearly half of patients with primary APS had an associated MTHFR mutation [5]. However, the simultaneous presence of all three prothrombotic factors and their compounded impact on pregnancy outcomes has not been documented in the literature to date.

The interaction between these genetic thrombophilias and autoimmune disorders such as APS remains poorly characterized, especially concerning prognostic implications and optimal therapeutic strategies. Theoretically, this triad can synergistically increase thrombotic risk. In women of childbearing age, this combination may lead to catastrophic obstetric outcomes such as eclampsia, placental insufficiency, and fetal loss [6,7]. We report the case of a young female patient who presented following eclampsia and subsequent neonatal loss, in whom thrombophilia screening revealed the presence of this triad.

## Case presentation

A 23-year-old previously healthy female smoker presented to the emergency department with a hypertensive emergency at 36 weeks of gestation. On arrival, her blood pressure was above 170/100 mmHg. The patient reported visual disturbances, severe nausea and vomiting, and headaches, which culminated in three seizures. The seizures were controlled with pharmacotherapy, followed by an urgent cesarean section. Unfortunately, the neonate died seven days after birth.

The patient was subsequently diagnosed with eclampsia and was referred to our institution for further evaluation several months later, after her obstetrician advised testing for a possible autoimmune cause of the episode due to the patient’s family history. No significant systemic symptoms were noted at the time. Family history included a grandparent with rheumatoid arthritis and interstitial lung disease, as well as a cousin with recurrent miscarriages.

Postpartum thrombophilia testing revealed primary APS, with positive lupus anticoagulant, anticardiolipin antibodies, and anti-β2-glycoprotein I antibodies, confirmed on two occasions spaced three months apart. Elevated antinuclear antibodies (ANA) were also detected (Table 1). Additionally, a heterozygous factor V Leiden mutation and a homozygous MTHFR mutation were identified. Treatment with hydroxychloroquine, aspirin, and low-molecular-weight heparin was initiated. The patient was counseled that any future pregnancies would be considered high-risk and require close monitoring to prevent adverse outcomes.

**Table 1:** Immunological and thrombophilic tests

Test (unit)	First test (December 2024)	Interpretation	Second test (February 2025)	Normal range	Interpretation
Anti-nuclear antibodies	-	-	1/320, fine speckled	<1/100	Positive
Lupus anticoagulant (qualitative)	Positive	-	Positive	-	Positive
Anti-cardiolipin IgG (GPU/mL)	122	Positive	120	<12	Positive
Anti-cardiolipin IgM (MPU/mL)	>120	Positive	80	<12	Positive
Anti-double stranded DNA IgG (IU/mL)	47	Negative	104	<100	Borderline
Rheumatoid factor (IU/mL)	-	-	11	<14	Negative
Anti-beta-2-glycoprotein I IgG (AU/mL)	32	Positive	>200	<20	Positive
Anti-beta-2-glycoprotein I IgM (AU/mL)	76.7	Positive	75	<20	Positive
Anti-cyclic citrullinated peptide (IU/mL)	7	Negative	<7	<17	Negative
Complement C3 (mg/dL)	-	-	110	90–180	Normal
Complement C4 (mg/dL)	-	-	31	10–40	Normal
Extractable nuclear antigen panel (qualitative)	Anti-mitochondrial antibody M2 (+++)		Negative	Negative	Negative
Factor V Leiden 1691 gene assay	Heterozygous mutation G > A	-	Heterozygous mutation G > A	Wild type GG	-
Methylenetetrahydrofolate reductase 1298 assay	Homozygous mutation A > C	-	Homozygous mutation A > C	Wild type AA	-
Homocysteine (μmol/L)	-	-	7	0-15	Normal

## Discussion

To our knowledge, this is the first reported case of a young woman with primary APS, heterozygous factor V Leiden mutation, and homozygous MTHFR mutation presenting with eclampsia and neonatal loss. Although the coexistence of APS with either factor V Leiden or MTHFR mutations has been previously described, the simultaneous presence of all three prothrombotic factors and their compounded impact on pregnancy outcomes has not been documented in the literature to date.

The laboratory results showing sustained positivity for all markers of primary APS conferred a markedly higher risk of obstetrical complications in our patient, necessitating a more stringent treatment approach and close follow-up for future events. This increased risk is supported by a study by Laurent et al. [8], who examined a cohort of 204 French patients with APS, among whom 68 were triple positive. Those with triple positivity had significantly higher rates of obstetric APS (45.6% versus 26.5%) and placental complications (17.6% versus 2.9%) compared to patients with double or single positivity. While the overall rate of thrombotic APS was similar between groups, triple-positive patients experienced a higher relapse rate (63.2% versus 39.7%) [8]. Additionally, the sixfold fluctuation in anti-β2GP IgG levels over the 12-week testing period (Table 1), although significant and indicative of an ongoing immune activity, does not correlate with an increased risk of clinical events, as documented by Frodlund et al. [9].

The measured plasma homocysteine level in the second set of tests in our patient was normal; however, the patient was on continuous folate supplementation from the prenatal period through testing. This supplementation likely contributed to the normal homocysteine level, consistent with findings by Bošković et al., who reported that folic acid supplementation is associated with lower plasma homocysteine levels [10].

Furthermore, the presence of a positive ANA with a speckled pattern and anti-dsDNA antibodies may indicate an underlying evolving autoimmune process that could culminate in a diagnosis of systemic lupus erythematosus (SLE), given their known association with SLE [11]. However, the patient currently exhibits no symptoms suggestive of SLE, whether articular, mucocutaneous, or visceral. Nonetheless, she requires close follow-up and clinical evaluation for any emerging manifestations of SLE. 

Primary APS is an autoimmune hypercoagulable disorder characterized by antiphospholipid antibodies that promote both arterial and venous thrombosis and are well-recognized causes of pregnancy morbidity, including preeclampsia, fetal loss, and placental insufficiency [12]. Antiphospholipid antibodies activate endothelial cells, monocytes, and platelets, causing increased expression of tissue factor and thromboxane A2, thereby promoting a procoagulant state. In addition, activation of the complement cascade may further drive thrombosis and fetal loss, especially when triggered by additional risk factors such as smoking. Antiphospholipid antibodies also interfere with proteins involved in clotting regulation, including prothrombin, factor X, proteins C and S, plasmin, and tissue factor pathway inhibitor, which hinders the inactivation of procoagulant factors and impairs fibrinolysis [13]. This case exemplifies these severe obstetric complications, underscoring the need for careful multidisciplinary management to improve future pregnancy outcomes.

Factor V Leiden mutation is a common inherited thrombophilia that causes resistance to activated protein C, thereby increasing the risk of VTE and pregnancy loss, particularly when combined with other prothrombotic conditions. Mutations in the MTHFR gene impair folate metabolism, leading to hyperhomocysteinemia, endothelial dysfunction, and a prothrombotic state. Although the individual contributions of APS, factor V Leiden, and MTHFR mutations to thrombosis and obstetric complications are well documented, their simultaneous presence in the same patient remains rare and poorly characterized.

Compared to previously published reports, this case uniquely illustrates the coexistence of primary APS with both factor V Leiden and MTHFR mutations in a young female patient presenting with severe obstetric complications. While factor V Leiden mutation has been variably reported in APS cohorts, with some studies indicating only a modest increase in thrombotic risk and others demonstrating a significant association, especially in patients positive for lupus anticoagulant, with an estimated 7.3-fold increase [12-14]. The simultaneous presence of MTHFR mutations remains scarcely documented. Notably, the synergistic prothrombotic effect of combined factor V Leiden and MTHFR mutations has been described in non-APS populations, where their concurrence markedly elevates VTE risk. However, few reports have addressed the integration of this genetic combination within the context of APS. One prior case highlighted recurrent coronary thrombosis in a patient with APS and factor V Leiden mutation, describing the compounded thrombotic risk [15]. Our case further extends this paradigm by adding a homozygous MTHFR mutation, thus representing a rare triad that has likely amplified both thrombotic and obstetrical risks beyond those observed in isolated APS or single-gene thrombophilia carriers.

Evidence also suggests that the burden of thrombophilia may influence the immunologic environment of the placenta and pregnancy, exacerbating endothelial dysfunction and inflammatory responses [16,17]. This underscores the need for comprehensive thrombophilia screening in patients with APS, particularly those with severe or recurrent pregnancy-related morbidities [12,18]. Early detection of thrombophilic risk factors through detailed and expanded workup allows for precise risk stratification and facilitates the implementation of individualized multidisciplinary therapeutic strategies, ultimately leading to improved patient outcomes. In this case, treatment with hydroxychloroquine, aspirin, and anticoagulation with low-molecular-weight heparin aligns with current recommendations aimed at mitigating the compounded thrombotic risk and improving maternal and fetal outcomes [12,18].

## Conclusions

By providing detailed clinical, immunological, and genetic characterization of this triad, our report contributes novel evidence to the evolving understanding of complex thrombophilia and immunogenicity. This case exemplifies the broad and heterogeneous autoimmune interplay and its impact on patients. Additionally, the presence of positive ANA and anti-dsDNA antibodies indicates the need for closer follow-up to detect any emerging SLE and its implications for personalized patient management. This case should encourage physicians to broaden their initial screening laboratory tests and consider implementing more comprehensive testing in patients who present with early and severe pre-eclampsia. Further studies are warranted to elucidate the prevalence, pathophysiological interactions, and optimal therapeutic approaches for patients harboring this rare combination of prothrombotic abnormalities, particularly female patients presenting with obstetrical or thrombotic events.
